# Do Race, Insurance Status, and Income Factors Impact Pathologic Fracture Presentation and Management?

**DOI:** 10.1002/cam4.71201

**Published:** 2025-09-22

**Authors:** Ashley B. Bozzay, Kara Churovich, Julio A. Rivera, Benjamin K. Potter

**Affiliations:** ^1^ Department of Surgery Uniformed Services University of the Health Sciences – Walter Reed National Military Medical Center Bethesda Maryland USA; ^2^ Orthopaedic Service, Department of Surgery Memorial Sloan Kettering Cancer Center New York USA; ^3^ Henry M. Jackson Foundation for the Advancement of Military Medicine Inc. Bethesda Maryland USA

**Keywords:** care management, metastatic bone disease, pathological fracture, socioeconomic factors

## Abstract

**Introduction:**

Metastatic bone disease (MBD) and pathologic fractures (PF) can impact quality of life, functionality, and survival. Understanding the management of PF and access to care and treatment in the U.S. healthcare system can improve patient outcomes, directly impacting treatment eligibility and overall survival. We ask: (1) Do race, income, and insurance status differ between prophylactic stabilization of impending and acute fixation of overt PF, respectively? (2) Are race, income, and insurance status associated with complications in patients with metastatic bone disease? (3) Are race, income, and insurance associated with length of stay and insurance type in patients with MBD?

**Methods:**

The NIS HCUP database was queried from 2016 to 2020 for pathological fractures. We then identified patients with associated cancer ICD‐10 diagnostic codes for the hospitalization (breast, prostate, renal, lung, thyroid, bone, and other). General linear models (GLMs) were used to answer each clinical question. We conducted a post hoc analysis of the other malignancies associated with osseous metastasis and performed the same studies as above.

**Results:**

We found 2050 prophylactically stabilized impending and 4181 acute fractures. There were no differences among races, income, or insurance status and the prevalence of impending versus acute fractures. We found that Black patients were more likely to have complications, while Hispanic patients were the least likely, compared to other races. Black patients had higher hospital costs, while both Black and Hispanic patients had more extended hospitalizations and were more likely to have Medicaid or no insurance compared to other races. Our post hoc analyses found that the rate of impending and acute fractures differed among race, income, and insurance status, depending on the metastatic cause.

**Conclusions:**

We identified healthcare disparities in patients with impending and overt PF due to MBD despite minimal to no differences in patient comorbidities, fracture management, or hospitalization complications.

## 1 Introduction

In 2023, 1,958,310 new cancer cases and 609,820 cancer deaths are projected to occur in the United States [1]. The advancements in oncologic medical treatments allow patients to live longer but increase their risk of developing metastatic disease complications [2]. Metastatic bone disease (MBD) is painful, and progression to pathologic fracture can impact quality of life, functional capacity, and survival [3]. Patients undergoing surgical intervention for impending versus completed pathological fracture have lower one‐year mortality rates and better outcomes (such as lower intraoperative blood loss and transfusions, decreased postoperative complications, and decreased reoperation rates) [4, 5].

Understanding the management of pathologic fractures and barriers to access to care and treatment in the United States can improve patient quality of life and functional status, which may also impact treatment eligibility and, thus, overall survival. Access to care is tightly linked to socioeconomic status, which is a multifaceted construct comprised of variables like insurance status, education, income, occupation, race, and even neighborhood‐level indicators. Specifically, income and insurance status impact disease‐specific survival in patients presenting with MBD from many of the most common carcinomas that metastasize to bone [6]. Surveillance, Epidemiology, and End Results (SEER) data have shown that the burden of MBD is higher in lower socioeconomic groups, suggesting these patients may have limited access to routine screening, delays in diagnosis related to healthcare costs, possible differences in subsequent cancer care delivery, and/or more advanced stages of disease at the time of diagnosis [7].

Moreover, MBD has been shown to differ among races. Jawad et al. [8] found that non‐Hispanic Black patients had higher rates of MBD originating in the prostate and breast compared to other races. At the same time, Native Americans and Alaskan Natives were more likely to have MBD originating from the kidneys and colon. Race and ethnicity have also been found to be significant predictors of the quality of health care and even postoperative adverse events, with minorities experiencing longer delays in care and limited access to vital procedures [9, 10, 11]. For example, one study found that Black patients had to wait 3 h longer to be imaged and had to wait 51% longer for surgical repairs compared to their White counterparts [12]. These types of delay may be responsible for worse postoperative outcomes in minorities and may lead to poorer quality of life, decreased function, and worse survival [9, 11]. Furthermore, others have found that race plays a role in fixation after fractures where Black patients had longer wait times, higher rates of early adverse events, and unplanned readmissions versus White patients [10, 13].

Therefore, the purpose of this study was to answer the following questions:

1. Do income, insurance status, and race differ between prophylactic stabilization of impending and acute fixation of overt pathologic fractures?
2. In patients with MBD, are race, income, and insurance status associated with complications, including death, and total cost of hospital stay?
3. Are race, income, and insurance status associated with length of stay and insurance type in patients with MBD?

## 2 Materials and Methods

### 2.1 Data Source

This retrospective study followed the Reporting of studies Conducted using Observational Routinely collected Data (RECORD) extension of the Strengthening the Reporting of Observational Studies in Epidemiology (STROBE) guidelines to ensure accuracy and completeness in observational study reporting [14, 15]. For the analysis, we utilized the National Inpatient Sample (NIS), Healthcare Cost and Utilization Project (HCUP), and Agency for Healthcare Research and Quality (AHRQ). NIS is a 20% representative sampling of all inpatient hospital encounters in the United States and is designed to represent healthcare use overall in the United States. The NIS contains clinical information that are typical in discharges including ICD‐9 and ICD‐10 codes for diagnoses, procedures, and external cause of injuries, patient characteristics (e.g., sex, age, race), hospital characteristics, expected payment source, total charges, discharge status, length of stay, and severity and comorbidity measures. NIS is functional for performing descriptive studies, deriving national estimates, cost analyses, rare disease analyses, and understanding trends over time [16]. There are approximately 7 million entries per year. HCUP is a limited healthcare dataset and does not require institutional review board review. We strictly adhered to Methodological Standards in Research Using the National Inpatient Sample [15, 16].

### 2.2 Study Population

**FIGURE 1:**
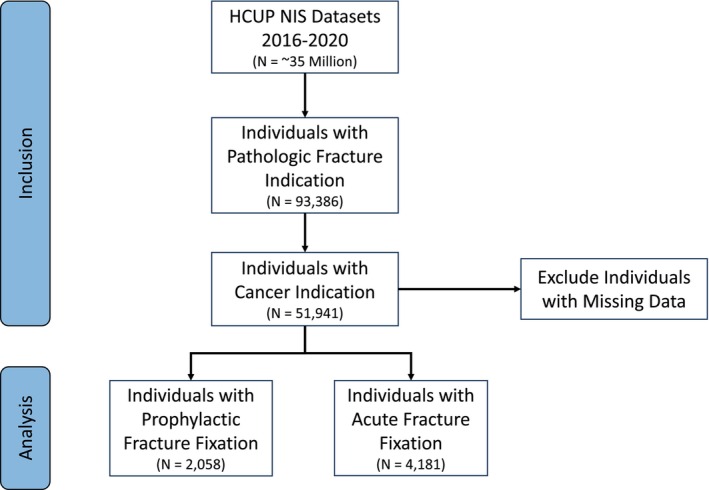
Strengthening the reporting of observational studies in epidemiology (STROBE) study design. HCUP, healthcare cost and utilization project; NIS, National inpatient sample.

We queried the NIS database from 2016 to 2020. We identified all patients with fracture diagnoses using the ICD‐10 parent code M84 diagnostic codes. We then identified patients with associated cancer ICD‐10 diagnostic codes for hospitalization (Appendix S1). This cohort was then stratified by prophylactic (impending fracture) or acute (completed fracture) stabilization for the hospitalization encounter using ICD‐10 procedure codes (Figure 1). Patient demographics, hospitalization encounter features, and the most common MBD pathologies were documented (Table 1).

**TABLE 1:** Demographics, cancers, and comorbidities by race of pathologic prophylactic and acute fractures used in this study.

Category	White		Black		Hispanic		Asian/Pacific Islander		Native American		Other	
	Prophylactic	Acute	Prophylactic	Acute	Prophylactic	Acute	Prophylactic	Acute	Prophylactic	Acute	Prophylactic	Acute
Sex												
Male	624 (30.32%)	1394 (33.34%)	114 (5.54%)	207 (4.95%)	69 (3.35%)	141 (3.37%)	21 (1.02%)	53 (1.27%)	1 (0.05%)	13 (0.31%)	20 (0.97%)	49 (1.17%)
Female	870 (42.27%)	1710 (40.90%)	174 (8.45%)	309 (7.39%)	99 (4.81%)	173 (4.14%)	26 (1.26%)	63 (1.51%)	4 (0.19%)	8 (0.19%)	36 (1.75%)	61 (1.46%)
Age												
< 55	205 (9.96%)	348 (8.62%)	61 (2.96%)	108 (2.58%)	52 (2.53%)	63 (1.51%)	17 (0.83%)	18 (0.43%)	1 (0.05%)	2 (0.05%)	10 (0.49%)	17 (0.41%)
55–64	395 (19.19%)	740 (17.80%)	103 (5.00%)	151 (3.61%)	49 (2.38%)	89 (2.13%)	10 (0.49%)	40 (0.96%)	1 (0.05%)	8 (0.19%)	17 (0.83%)	31 (0.74%)
65–74	467 (22.69%)	1007 (24.09%)	76 (3.69%)	136 (3.25%)	44 (2.14%)	87 (2.08%)	17 (0.83%)	32 (0.77%)	1 (0.05%)	5 (0.12%)	16 (0.78%)	31 (0.74%)
75–84	323 (15.69%)	708 (16.93%)	37 (1.80%)	91 (2.18%)	20 (0.97%)	51 (1.22%)	3 (0.15%)	16 (0.38%)	1 (0.05%)	3 (0.07%)	12 (0.58%)	24 (0.57%)
85+	104 (5.05%)	301 (7.20%)	11 (0.53%)	30 (0.72%)	3 (0.15%)	24 (0.57%)	—	10 (0.24%)	1 (0.05%)	3 (0.07%)	1 (0.05%)	7 (0.17%)
Income												
$1–$49,999	331 (16.08%)	728 (17.41%)	150 (7.29%)	267 (6.39%)	56 (2.72%)	112 (2.68%)	6 (0.29%)	11 (0.26%)	3 (0.15%)	4 (0.10%)	12 (0.58%)	28 (0.67%)
$50,000–$64,999	364 (17.69%)	830 (19.85%)	51 (2.48%)	103 (2.46%)	45 (2.19%)	93 (2.22%)	11 (0.53%)	22 (0.53%)	—	6 (0.14%)	16 (0.78%)	27 (0.65%)
$65,000–$85,999	417 (20.26%)	782 (18.70%)	49 (2.38%)	90 (2.15%)	40 (1.94%)	61 (1.46%)	6 (0.29%)	31 (0.74%)	1 (0.05%)	9 (7.15%)	8 (0.39%)	28 (0.67%)
$86,000+	382 (18.5%)	764 (18.27%)	38 (1.85%)	56 (1.34%)	27 (1.31%)	48 (1.15%)	24 (1.17%)	52 (1.24%)	1 (0.05%)	2 (0.05%)	20 (0.97%)	27 (0.65%)
Insurance												
Medicare	890 (43.35%)	2010 (48.07%)	142 (6.90%)	281 (6.72%)	62 (3.01%)	151 (3.61%)	19 (0.92%)	47 (1.12%)	4 (0.19%)	11 (0.26%)	31 (1.51%)	60 (1.44%)
Medicaid	100 (4.86%)	222 (5.31%)	52 (2.53%)	91 (2.18%)	44 (2.14%)	58 (1.39%)	9 (0.44%)	18 (0.43%)	—	5 (0.12%)	6 (0.29%)	18 (0.43%)
Private insurance	448 (21.77%)	740 (17.70%)	78 (3.79%)	102 (2.44%)	46 (2.24%)	73 (1.75%)	17 (0.83%)	41 (0.98%)	—	5 (0.12%)	17 (0.83%)	26 (0.62%)
Self‐pay	13 (0.63%)	56 (1.34%)	9 (0.44%)	21 (0.50%)	12 (0.58%)	16 (0.38%)	—	7 (0.17%)			2 (0.10%)	3 (0.07%)
No charge	1 (0.05%)	2 (0.05%)	1 (0.05%)	1 (0.02%)	1 (0.05%)	—	—	—	—	—	—	—
Other	42 (2.04%)	74 (1.77%)	6 (0.29%)	20 (0.48%)	3 (0.15%)	16 (0.38%)	2 (0.10%)	3 (0.07%)	1 (0.05%)	—	—	3 (0.07%)
Cancer												
Breast	208 (10.11%)	429 (10.26%)	40 (1.94%)	65 (1.55%)	30 (1.46%)	46 (1.10%)	3 (0.15%)	19 (0.45%)	—	1 (0.02%)	7 (0.34%)	18 (0.43%)
Prostate	87 (4.23%)	231 (5.52%)	29 (1.41%)	40 (0.96%)	12 (0.58%)	20 (0.48%)	1 (0.05%)	11 (0.26%)	—	—	6 (0.29%)	6 (0.14%)
Renal	95 (4.62%)	184 (4.40%)	13 (0.63%)	18 (0.43%)	13 (0.63%)	18 (0.43%)	1 (0.05%)	6 (0.14%)	—	4 (0.10%)	2 (0.10%)	2 (0.05%)
Lung	332 (16.13%)	572 (13.68%)	38 (1.85%)	70 (1.67%)	17 (0.83%)	32 (0.77%)	12 (0.58%)	19 (0.45%)	1 (0.05%)	2 (0.05%)	8 (0.39%)	21 (0.50%)
Thyroid	4 (0.19%)	13 (0.31%)	3 (0.15%)	7 (0.17%)	—	3 (0.07%)	—	4 (0.10%)	—	—	1 (0.05%)	—
Bone	21 (1.02%)	67 (1.60%)	5 (0.24%)	7 (0.17%)	8 (0.39%)	9 (7.15%)	6 (0.29%)	1 (0.02%)	—	—	—	2 (0.05%)
Other^a^	747 (36.30%)	1608 (38.46%)	160 (7.77%)	309 (7.39%)	88 (4.28%)	186 (4.45%)	27 (1.31%)	56 (1.34%)	4 (0.19%)	14 (0.33%)	32 (1.55%)	61 (1.46%)
Comorbidity												
Anemia	411 (19.97%)	1000 (23.92%)	129 (6.27%)	243 (5.81%)	62 (3.01%)	114 (2.73%)	11 (0.53%)	34 (0.81%)	2 (0.10%)	6 (0.14%)	14 (0.68%)	40 (0.96%)
Congestive heart failure	137 (6.66%)	336 (8.04%)	27 (1.31%)	58 (1.39%)	8 (0.39%)	28 (0.67%)	2 (0.10%)	9 (7.15%)	1 (0.05%)	2 (0.05%)	5 (0.24%)	10 (0.24%)
Coronary artery disease	49 (2.38%)	94 (2.25%)	7 (0.34%)	22 (0.53%)	7 (0.34%)	4 (0.10%)	1 (0.05%)	1 (0.02%)	—	—	1 (0.05%)	4 (0.10%)
Diabetes mellitus	314 (15.26%)	660 (15.79%)	66 (3.21%)	149 (3.56%)	41 (1.99%)	109 (2.61%)	18 (0.87%)	39 (0.93%)	4 (0.19%)	6 (0.14%)	10 (0.49%)	28 (0.67%)
Obesity	349 (16.96%)	783 (18.73%)	74 (3.60%)	158 (3.78%)	44 (2.14%)	73 (1.75%)	8 (0.39%)	23 (0.55%)	2 (0.10%)	4 (0.10%)	7 (0.34%)	31 (0.74%)
Hypothyroidism	218 (10.59%)	497 (11.89%)	16 (0.78%)	40 (0.96%)	17 (0.83%)	31 (0.74%)	4 (0.19%)	17 (0.41%)	3 (0.15%)	3 (0.07%)	7 (0.34%)	10 (0.24%)
Kidney disease	167 (8.11%)	448 (10.72%)	49 (2.38%)	106 (2.54%)	18 (0.87%)	50 (1.20%)	5 (0.24%)	21 (0.50%)	1 (0.05%)	5 (0.12%)	5 (0.24%)	16 (0.38%)
Depression	190 (9.23%)	424 (10.14%)	25 (1.21%)	45 (1.08%)	13 (0.63%)	23 (0.55%)	2 (0.10%)	4 (0.10%)	1 (0.05%)	2 (0.05%)	6 (0.29%)	12 (0.29%)
Hypertension	740 (35.96%)	1609 (38.48%)	171 (8.31%)	9 (7.15%)	78 (3.79%)	146 (3.49%)	19 (0.92%)	63 (1.51%)	3 (0.15%)	11 (0.26%)	25 (1.21%)	61 (1.46%)
Peripheral vascular disease	63 (3.06%)	178 (4.26%)	11 (0.53%)	31 (0.74%)	4 (0.19%)	17 (0.41%)	1 (0.05%)	6 (0.14%)	—	4 (0.10%)	2 (0.10%)	3 (0.07%)
Chronic obstructive pulmonary disease	309 (15.01%)	683 (16.34%)	50 (2.43%)	86 (2.06%)	13 (0.63%)	41 (0.98%)	7 (0.34%)	15 (0.36%)	2 (0.10%)	5 (0.12%)	8 (0.39%)	27 (0.65%)
Liver disease	58 (2.82%)	125 (2.99%)	14 (0.68%)	28 (0.67%)	8 (0.39%)	14 (0.33%)	5 (0.24%)	7 (0.17%)	—	1 (0.02%)	4 (0.19%)	3 (0.07%)

*Note:* Values outside the parentheses represent sample size while values in the parentheses indicate the proportion of the sample.

^a^ Other included CD‐10 code range for Malignant neoplasms C00 to C96: Malignant neoplasm lip, oral cavity, pharynx; neuroendocrine tumors; Malignant neoplasm of digestive organs; Malignant neoplasms of respiratory and intrathoracic organs; Melanoma and other malignant neoplasms of skin Malignant neoplasms of mesothelial and soft tissue; Malignant neoplasms of female genital organs; Malignant neoplasms of male genital organs; Malignant neoplasms of urinary tract; Malignant neoplasms of eye, brain and other parts of central nervous system; Malignant neoplasms of thyroid and other endocrine glands; Malignant neoplasms of ill‐defined, other secondary and unspecified sites; Malignant neoplasms of lymphoid, hematopoietic and related tissue; Malignant neoplasms of ill‐defined, other secondary and unspecified sites.

### 2.3 Variables

Hospital admissions were characterized by race, socioeconomic factors (insurance type, median household income), clinical factors (comorbidities, cancer type), and hospital characteristics (length of stay and total hospital charges). Comorbidities (Table 1) and complications (Table 2) were assessed based on the associated hospitalization ICD‐10 diagnostic code (Table 1). Variables of interest included race (White, Black, Hispanic, Asian/Pacific Islander, Native American, and other), insurance type (Medicare, Medicaid, private, self‐pay, no charge, and other), median household income, primary cancer type (breast, prostate, renal, lung, thyroid, bone, and other), comorbidities, and complications. Entries with missing values were excluded from the analysis. The outcome of interest included hospitalization complications, length of stay, and total hospital charges.

**TABLE 2:** Complications following pathologic fractures by race where values outside the parentheses indicate sample size and values in the paratheses indicate the proportion they comprise.

	White		Black		Hispanic		Asian/Pacific Islander		Native American		Other	
	Prophylactic	Acute	Prophylactic	Acute	Prophylactic	Acute	Prophylactic	Acute	Prophylactic	Acute	Prophylactic	Acute
Acute anemia	332 (16.13%)	931 (22.27%)	68 (3.30%)	155 (3.71%)	33 (1.60%)	79 (1.89%)	13 (0.63%)	29 (0.69%)	2 (0.10%)	7 (0.17%)	13 (0.63%)	35 (0.84%)
Surgical site infection	2 (0.10%)	2 (0.05%)	1 (0.05%)	—	—	—	—	—	—	—	—	—
Wound dehiscence	1 (0.05%)	3 (0.07%)	—	—	—	—	1 (0.05%)	—	—	—	—	1 (0.02%)
Acute renal failure	163 (7.92%)	455 (10.88%)	55 (2.67%)	107 (2.56%)	19 (0.92%)	27 (0.65%)	2 (0.10%)	12 (0.29%)	—	1 (0.02%)	6 (0.29%)	19 (0.45%)
Acute myocardial infarction	12 (0.58%)	30 (0.72%)	2 (0.10%)	5 (0.12%)	—	5 (0.12%)	—	—	—	—	—	2 (0.05%)
Pneumonia	75 (3.64%)	134 (3.20%)	20 (0.97%)	19 (0.45%)	8 (0.39%)	16 (0.38%)	—	4 (0.10%)	1 (0.05%)	—	3 (0.15%)	6 (0.14%)
Acute heart failure	134 (6.51%)	331 (7.92%)	27 (1.31%)	57 (1.36%)	8 (0.39%)	28 (0.67%)	2 (0.10%)	9 (0.22%)	1 (0.05%)	2 (0.05%)	5 (0.24%)	10 (0.24%)
Pulmonary embolism	33 (1.60%)	64 (1.53%)	4 (0.19%)	20 (0.48%)	8 (0.39%)	8 (0.19%)	1 (0.05%)	2 (0.05%)	—		2 (0.10%)	1 (0.02%)
Mortality	43 (2.09%)	86 (2.06%)	8 (0.39%)	12 (0.29%)	4 (0.19%)	4 (0.10%)	2 (0.10%)	4 (0.10%)	—	2 (0.05%)	1 (0.05%)	6 (0.14%)

### 2.4 Statistical Methods

To answer our clinical questions, we used generalized linear models to determine if race, income, and insurance status differed by prophylactic and acute fixations cohorts, with a two‐tailed alpha of < 0.05. We then used the same approach to determine if race, income, and insurance characteristics were correlated with the risk of all complications. We then repeated this analysis, stratifying by each complication type for complications that had more than 10 total occurrences [17]. We also used generalized linear models to determine if race or median household income correlated with length of hospitalization, total hospital charges, or insurance type. All analyses were conducted using R Statistical Software [18].

### 2.5 Post Hoc Analyses

In addition to our primary analyses, we used generalized linear models to address whether race, income, and insurance status differed among the “other” cancer categories (Appendix S1), as this group comprised a substantial proportion of total patients from the study population. We tested cancers with more than 30 occurrences and used a two‐tailed alpha of < 0.05.

## 3 Results

### 3.1 Do Race, Income, and Insurance Status Differ Among Prophylactic and Acute Fixations?

We found 2050 prophylactically stabilized impending fractures and 4181 acute fractures for 6239 pathologic fracture stabilizations (Table 1). There were no differences among races; income and insurance status did not affect the prevalence of impending versus acute fractures.

### 3.2 In Patients With Metastatic Bone Disease, Are Race, Income, and Insurance Status Associated With Complications (Including Death) and Total Cost of Hospital Stay?

Using generalized linear models, we found that Black patients were more likely to have a complication, while Hispanic patients were the least likely to have a complication compared to other races (*p* = 0.002). There was no difference in complication rates based on income and insurance status. We then stratified our data by complication type and found that acute anemia was least prevalent in Hispanic individuals (*p* = 0.05) but more prevalent in the highest median household income (*p* = 0.01, $86,000+). We also found that Black individuals were more likely to have acute renal failure (*p* < 0.001) as a complication. At the same time, Hispanic and Asian people were less likely to have acute renal failure (*p* = 0.02). Hispanics were also less likely to have acute heart failure (*p* = 0.05), as were individuals from 2nd ($50,000–$64,999) and 4th ($86,000+) median household income quartiles. We did not find a difference between race or income and insurance status and acute myocardial infarction, pneumonia, or mortality. In terms of total hospital charges, we found that Black (*p* < 0.001, mean = $27,031) and Hispanic (*p* < 0.001, mean = $50,185) patients were charged the most, as were patients who made over $86,000 annually (*p* < 0.001).

### 3.3 In Patients With Metastatic Bone Disease, Are Race, Income, and Insurance Status Associated With Length of Stay and Insurance Type?

**FIGURE 2:**
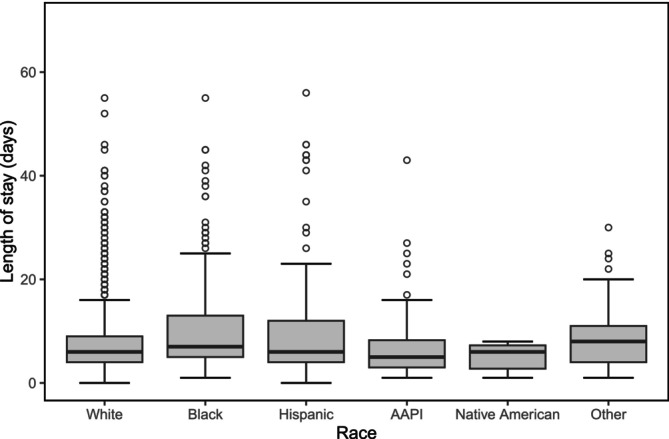
Difference in length of hospital stay by race. AAPI, Asian/Pacific Islander.

We found a positive correlation between length of stay and race (Figure 2). Specifically, Black (*p* < 0.001) and Hispanic (p < 0.001) patients had more extended hospitalizations when compared to other races. Similarly, we found that individuals from the 2nd median household income quartile ($50,000–$64,999) were also more likely to spend more time in the hospital than other median household incomes (*p* = 0.04).

Lastly, we found a difference in insurance type by race, such that Black and Hispanic individuals were more likely to have Medicaid or no insurance (self‐pay). In contrast, White individuals either had Medicare or private insurance (*p* = 0.008). Our generalized linear model found that individuals with Medicaid or self‐paid/no insurance were more likely to have longer inpatient stays than other insurance types (*p* = 0.01).

### 3.4 Unexpected Findings

Additionally, we found that 4022 people had hospitalizations associated with pathologic fractures that were not related to breast, prostate, renal, lung, thyroid, or bone, making up roughly 64% of all fractures (4022/6239). The 4022 fracture cohort comprised various malignancies besides carcinoma (Appendix S1). Of these 4022 malignancy‐associated pathologic fractures, malignant neoplasms of ill‐defined, other secondary and unspecified sites comprised ~51% (2049/4022) and 34% (1369/4022) of the other malignancy category were associated with “Malignant neoplasm of lymphoid, hematopoietic and related tissue.”

We found individuals with pathologic fractures and malignant neoplasms that were ill‐defined were least prevalent in Black individuals (*p* < 0.001) and the “other” race category (*p* = 0.009). Also, pathologic fractures with malignant neoplasm of lymphoid, hematopoietic, and related tissue were substantially higher among Black (*p* < 0.001), Hispanic (*p* = 0.002), and “other” races (*p* = 0.01) as well as being highest among the lowest (*p* < 0.001, $1–$49,999) and second lowest (*p* = 0.02, $50,000–$64,999) socioeconomic status. Additionally, pathologic fractures with malignant neoplasm of digestive organs were least common among Black individuals (*p* = 0.04) but highest among people of the lowest socioeconomic status (*p* = 0.02, $1–$49,999).

## 4 Discussion

Many patients with metastatic cancer have skeletal involvement. Understanding the management of pathologic fractures as well as barriers to access to care from factors such as age, sex, race, income, and insurance status in the United States healthcare system can improve patient quality of life and functional status, which may impact survival. In an extensive nationwide database analysis, we found no differences in race or income and insurance status concerning the prevalence of impending versus acute fractures at the time of fixation. There were no differences in complication rates based on income and insurance status; however, Black patients were more likely to have a complication when compared to other races. Additionally, Black patients had both more prolonged and more costly hospitalizations when compared to other races.

## 5 Limitations

This study has inherent limitations. First, the HCUP NIS is a large administrative database (e.g., diagnostic code, procedure codes, and cost documentation) assembled from data intended for billing purposes. Therefore, many diagnostic procedures are underrepresented because there is no financial benefit associated with the intervention [19]. In our patient population, this was important because we assumed that stabilization procedures were analogous to patients presenting with impending pathologic fractures, while acute fixation procedures were analogous to patients presenting with completed pathologic fractures [20].

Although NIS is not necessarily a clinical database, it has been used to formulate clinical practice guidelines, quality improvement initiatives, and measures, assess the effectiveness of surgical techniques, identify healthcare disparities, and perform comparative effectiveness research [21]. Therefore, the strengths of NIS make it ideal for performing basic descriptive studies, deriving national estimates, and understanding trends over time for presentation, comorbidities, hospital stay complications (as NIS does not report 30‐day complications after discharge), and overall management of patients with pathologic fractures and associated early complications [21].

It is also essential to recognize relevant sociodemographic variables when researching healthcare disparities. A previous *CORR* editorial discussed the importance of assessing relevant sociodemographic variables using metrics such as the Distressed Communities Index, the Social Vulnerability Index, and the Area Deprivation Index before concluding that healthcare disparities are attributed to race [22]. These analyses were not feasible due to the limitations of the HCUP NIS database [22].

Overall, we recognize the inherent limitations of administrative data and the constraints of results that may impact healthcare decisions. However, we ensured adherence to Methodological Standards in Research Using the National Inpatient Sample and followed best practices previously when using NIS for research purposes [23, 24]. Additionally, we followed the *Clinical Orthopaedics and Related Research (CORR)* Standard for large‐database reviews to ensure our clinical questions of interest were appropriate for HCUP NIS and pertinent to readers [16, 21, 23, 25]. We believe that, despite these data limitations, our analysis was appropriate and our findings valid.

### 5.1 Do Race, Income, and Insurance Status Differ Among Prophylactic and Acute Fixations?

We found that Black individuals were marginally more likely to present with an impending pathologic fracture compared to other races. We did not find additional differences among races. Moreover, we did not find a difference between income and insurance status and the prevalence of prophylactic and acute fractures. This is not consistent with past publications, which conclude that ethnic minorities, as well as uninsured and Medicaid‐insured patients, have an increased risk of advanced‐stage cancers at the time of diagnosis [7]. However, a previous study using the 2002 to 2014 HCUP NIS database identified possible disparities in access to prophylactic treatment (with a higher percentage of white, male, and privately insured patients receiving prophylactic fixation) [20]. Our results from 2016 to 2020 may be a reflection of positive changes in nationwide oncology healthcare patterns or a reflection of the limitations in the database.

Additionally, the patient's comorbidity profiles were relatively similar. Therefore, overall, patient health demographic characteristics cannot account for the differences seen for age and insurance type. The acute fixation cohort did not have increased mortality, although this may have been due to the aforementioned limitations of the HCUP NIS. The complication profiles were relatively similar, except the acute fixation cohort had increased rates of acute anemia, acute renal failure, and acute heart failure. These findings are consistent with a previous publication that found no difference in hospitalization or 30‐day postoperative complication profiles for impending versus acute (completed) pathological fractures [4]. Furthermore, the 90‐day survival rate did not differ; however, one‐year survival [4] and overall survival [26] were worse in acute (completed) pathological fractures.

### 5.2 In Patients With Metastatic Bone Disease, Are Race, Income, and Insurance Status Associated With Complications (Including Death) and Total Cost of Hospital Stay?

Complication profiles were relatively similar between cohorts. However, overall, Black patients were more likely to have complications when compared to other races. Specifically, Black patients were more likely to have acute renal failure when compared to other racial groups. Black patients are more susceptible to chronic kidney disease, but recent consensus statements on perioperative acute renal disease do not list race as a risk factor for acute renal failure [27]. Additionally, we found Black and Hispanic individuals were marginally more likely to have a pulmonary embolism when compared to other races. A previous systematic review indicates patients undergoing surgical procedures for osseous metastatic disease have an increased perioperative risk of developing a venous thromboembolic event (VTE) [28]. Although we report a marginal racial difference, to our knowledge, no previous studies have identified race in the setting of a pathological fracture as a risk factor for VTE. Finally, in this nationwide database analysis, Black patients had more costly hospitalizations despite minimal differences in complication profiles. This may have been due to prolonged treatment of perioperative complications or difficulty with disposition at the time of discharge.

### 5.3 In Patients With Metastatic Bone Disease, Are Race, Income, and Insurance Status Associated With Length of Stay and Insurance Type?

We found a correlation between length of stay and race. Specifically, Black and Hispanic patients had more extended hospitalizations when compared to other races. This finding is consistent with increased total hospitalization cost charges for the same racial groups. This is especially important given that we found no difference in patient comorbidity and complication profiles. This may be attributed to insurance status and difficulty with disposition, as we did see Black and Hispanic individuals were more likely to have Medicaid or no insurance (self‐pay) than White patients. Previous publications from the same database used here (HCUP NIS) have reported similar findings [29]. The observed association between measures of high‐cost, minority race, and ethnicity groups despite no difference in care may reflect differences attributed to social support factors or even race itself. Healthcare systems should be cognizant of this when treating patients with MBD and provide improved case management infrastructure or access for this patient population [2].

### 5.4 Unexpected Findings

Unexpectedly, we found a large portion of patients presenting with pathologic fractures secondary to malignancies other than carcinoma. These may be de novo metastases due to other malignancies as patients live longer with improved cancer therapies or advancements in diagnostics [30]. In a previous epidemiology review of the SEER database, 18.8 per 100,000 de novo bone metastases were diagnosed per year from 2010 to 2015 [30, 31]. Although the majority of these were from lung and breast cancer, the authors also identified other pathologies such as neuroendocrine and gastrointestinal malignancies [30]. This is consistent with our findings. However, many pathologies were not classified by ICD‐10, which may be due to a lack of tissue diagnosis at the time of stabilization or fixation (e.g., initial presentation of cancer with advanced metastatic disease) or miscoding.

In our post hoc analysis, approximately 51% (2049/4022) of the other malignancy category was coded as “Malignant neoplasm that was ill‐defined.” It is reassuring that no racial, income, or insurance disparities were identified in this sizable unknown category. However, 34% (1369/4022) of the other malignancy category was also coded as “Malignant neoplasm of lymphoid, hematopoietic and related tissue.” The frequency of pathological fracture from lymphoid, hematopoietic, and related tissue in the entire cohort was 22% (1369/6329). We did find in this subgroup that Black and Hispanic patients, as well as lower socioeconomic categories, were more likely to present with any pathological fractures. The racial, income, and insurance findings in the remaining pathologies in the other category should be interpreted cautiously, as these represent small percentages of the overall patient cohort. Overall, it is essential to recognize the epidemiology of metastatic cancer is evolving through time and becoming more heterogeneous beyond the classic pathologies that metastasize to bone. Thus, further investigation of this cohort is warranted better to understand the metastatic propensity and behavior of malignancies as patients survive longer and assess the influence of patient sociodemographic variables in healthcare delivery and outcomes.

## 6 Conclusions

This study identified healthcare disparities and inequities in patients with pathologic fractures due to MBD despite minimal to no differences in patient comorbidities, fracture management, or hospitalization complications. Many studies about healthcare disparities in orthopedic surgery have attributed care findings to race without also considering potentially relevant sociodemographic variables, such as income and insurance status, in specific patient populations. At admission, patients should have access to resources to optimize patient care plans, including disposition and healthcare cost containment.

## Author Contributions

**Ashley B. Bozzay:** conceptualization (equal), data curation (equal), investigation (supporting), methodology (equal), supervision (supporting), validation (equal), visualization (equal), writing – original draft (lead), writing – review and editing (equal). **Kara Churovich:** data curation (equal), formal analysis (equal), visualization (equal), writing – review and editing (equal). **Julio A. Rivera:** conceptualization (supporting), data curation (supporting), formal analysis (lead), investigation (equal), methodology (supporting), project administration (supporting), resources (supporting), software (lead), supervision (supporting), validation (equal), visualization (equal), writing – original draft (equal), writing – review and editing (equal). **Benjamin K. Potter:** conceptualization (lead), investigation (supporting), project administration (lead), supervision (lead), supervision (lead), writing – original draft (supporting), writing – original draft (supporting), writing – review and editing (supporting), writing – review and editing (supporting).

## Ethics Statement

Under HIPAA, review by an institutional review board (IRB) is not required for use of limited data sets.

## Conflicts of Interest

The authors declare no conflicts of interest. The contents of this publication are the sole responsibility of the author(s) and do not necessarily reflect the views, opinions, or policies of Uniformed Services University of the Health Sciences (USUHS), The Henry M. Jackson Foundation for the Advancement of Military Medicine Inc., the Department of Defense (DoD) or the Departments of the Army, Navy, or Air Force. Mentioning trade names, commercial products, or organizations does not imply endorsement by the U.S. Government.

## Supporting information


**Appendix S1:** List of other metastatic cancers (*N* = 4022) and their prevalence within the full dataset (*N* = 6329).

## Data Availability

The data that support the findings of this study are openly available on https://hcup‐us.ahrq.gov/. The HIPAA Privacy Rule sets national standards for patient rights with respect to health information. This rule protects individually identifiable health information by establishing conditions for its use and disclosure by covered entities. HCUP Databases are Limited Data Sets. HCUP databases conform to the definition of a limited data set. A limited data set is healthcare data in which 16 direct identifiers, specified in the Privacy Rule, have been removed. Under HIPAA, review by an institutional review board (IRB) is not required for use of limited data sets. HCUP is consistent With HIPAA Regulations. The HCUP DUA is consistent with HIPAA requirements for use of a limited data set.
