# Supplementary material for: Do Race, Insurance Status, and Income Factors Impact Pathologic Fracture Presentation and Management?
Source: Cancer Med. 2025 Sep 22;14(18):e71201. doi: 10.1002/cam4.71201 (PMC12451829; doi:10.1002/cam4.71201)
Supplement: Supplementary file 1 — Appendix S1: List of other metastatic cancers (N = 4022) and their prevalence within the full dataset (N = 6329). [file CAM4-14-e71201-s001.docx]

Appendix 1.

List of other metastatic cancers (N=4022) and their prevalence within the full dataset (N=6329).

| Cancer type | N (%) |
| --- | --- |
| Malignant neoplasm that were ill-defined | 2049 (50.94%) |
| Malignant neoplasm of lymphoid, hematopoietic and related tissue | 1369 (33.81%) |
| malignant neoplasm of digestive organs | 290  (7.21%) |
| malignant neoplasm of female genital organs | 67  (1.67%) |
| malignant neoplasm of mesothelioma and soft tissue | 64  (1.59%) |
| malignant neoplasm of urinary tract | 59  (1.47%) |
| melanoma and other malignant neoplasm of skin | 56  (1.39%) |
| malignant neoplasm of lip, oral cavity, and pharynx | 30  (0.75%) |
| malignant neuroendocrine tumors, secondary neuroendocrine tumors | 29  (0.72%) |
| malignant neoplasm of thyroid and other endocrine glands | 7  (0.17%) |
| malignant neoplasms of respiratory and intrathoracic organs | 5  (0.12%) |
| malignant neoplasms of eye, brain and other parts of central nervous system | 5  (0.12%) |
| and malignant neoplasms of male genital organs | 1  (0.02%) |
